# Association Between Bone Density and Maxillary Canine Impaction: A CBCT-Based Study

**DOI:** 10.3390/jcm15020776

**Published:** 2026-01-18

**Authors:** Gianna Dipalma, Angelo Michele Inchingolo, Roberta Morolla, Francesco Inchingolo, Daniela Di Venere, Cinzia Maspero, Andrea Palermo, Grazia Marinelli, Alessio Danilo Inchingolo

**Affiliations:** 1 Department of Interdisciplinary Medicine, University of Bari “Aldo Moro”, 70124 Bari, Italy; roberta.morolla@uniba.it (R.M.); francesco.inchingolo@uniba.it (F.I.); daniela.divenere@uniba.it (D.D.V.); graziamarinelli@live.it (G.M.); alessiodanilo.inchingolo@uniba.it (A.D.I.); 2 Department of Biomedical, Surgical and Dental Sciences, Milan University, 20122 Milan, Italy; cinzia.maspero@unimi.it; 3 Fondazione IRCCS Cà Granda, Ospedale Maggiore Policlinico, 20100 Milan, Italy; 4 Department of Experimental Medicine, University of Salento, 73100 Lecce, Italy; andrea.palermo@unisalento.it

**Keywords:** TC Cone Beam, hounsfield index, impacted teeth, orthodontic treatment

## Abstract

**Background/Objective:** Maxillary canine impaction is a frequent orthodontic challenge. Three-dimensional CBCT assessment allows precise evaluation of periradicular bone density, which may play a role in eruption failure. This study aimed to compare bone density (HU) around impacted canines with that of the contralateral erupted tooth and to assess correlations with age and sex. **Methods:** A total of 26 patients (10 males, 16 females; 13–19 years) with unilateral maxillary canine impaction were examined. Pre-treatment CBCT scans acquired were analyzed. Bone density was measured in HU at three root levels (cervical, middle, apical) and in four regions (buccal, palatal, mesial, distal). Statistical analyses included Student’s *t*-tests, linear regression, and correlation coefficients, with significance set at *p* < 0.05. **Results:** Tooth 2.3 was the most frequently impacted (61.5%), with a slight predominance of palatal impactions (53.8%). Bone density was significantly higher around impacted canines than around the contralateral erupted teeth in all regions and levels (*p* = 0.000), with values increasing from the cervical portion toward the apex. In impacted canines, bone density increased significantly with age, whereas no meaningful differences were found between males and females. **Conclusions:** Higher bone density surrounding impacted canines supports its potential role in eruption failure. The age-related increase highlights the clinical importance of early diagnosis and timely orthodontic–surgical intervention.

## 1. Introduction

Nowadays, clinical and radiographic evidence of teeth that are impacted or positioned abnormally within the bone is becoming increasingly common [1,2,3,4,5,6,7]. A tooth is considered impacted when it has not erupted into the dental arch even though the root has fully developed, or when its eruption occurs more than six months after that of the contralateral tooth, provided the root is completely formed [8,9,10,11,12]. From an epidemiological standpoint, the teeth most frequently affected are the lower third molars, followed by the maxillary canines [13,14,15,16,17,18,19,20,21]. In fact, the prevalence of impacted canines reported in the literature ranges roughly from 0.2% to 2.8% [22,23,24,25,26,27,28,29,30]. Managing these teeth can be challenging, and treatment is only truly successful when the guided eruption and subsequent alignment allow the tooth to reach a proper position in the arch while maintaining healthy periodontal tissues [31,32,33,34,35,36,37,38,39,40,41,42]. To minimize the risk of bone dehiscence or unfavorable orthodontic and esthetic outcomes, the treatment plan should, as far as possible, reproduce the tooth’s natural eruption pathway—ideally toward the center of the alveolar ridge—as suggested by several authors [24,43,44,45,46,47,48,49].

Because many factors influence both orthodontic and periodontal outcomes, treating impacted canines requires a multidisciplinary approach [14,50,51,52,53,54,55,56,57].

**Figure 1:**
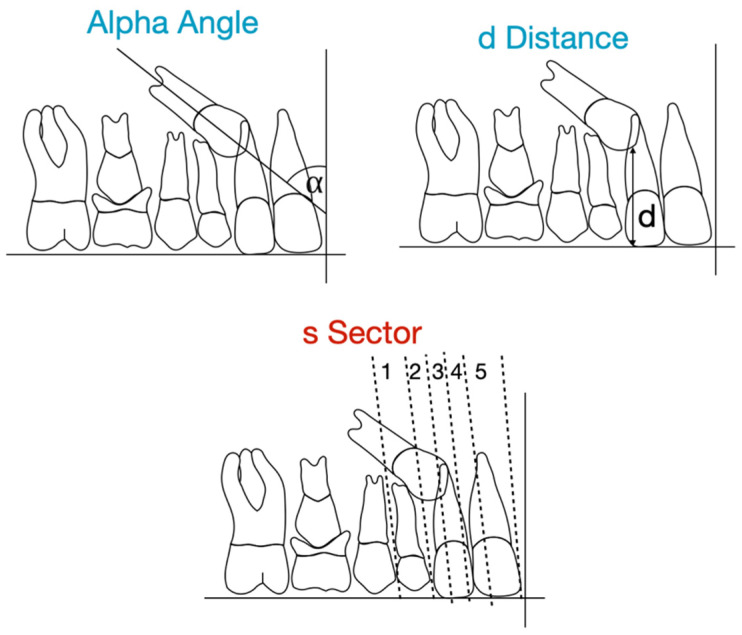
Method described by Ericson and Kurol: α–angle, the angle between the long axis of the canine and the midline; d–distance, the distance in millimeters from the canine cusp tip to the occlusal plane; and sector, the mesiodistal crown position classified from sector 1 to 5.

Among these factors, certain pre-treatment radiographic features—such as the “angle α,” the “distance d,” and the sector of impaction described by Ericson and Kurol—have proven useful as predictive indicators [58]. They can help estimate the time needed for orthodontic traction as well as the overall duration of orthodontic treatment aimed at bringing the tooth into its correct position (Figure 1).

### 1.1. Palatal or Vestibular Inclusion of the Impacted Canine

The crown of the permanent maxillary canine is fully formed by the age of 6–7 and erupts in the arch between the ages of 11 and 13 [59,60,61,62,63,64].

It has been estimated that the eruption path of the canine extends over a distance of more than 22 mm [65,66,67,68,69,70,71]. Precisely because of this prolonged eruption path, the canine can become displaced in two main directions: palatal or vestibular [72,73,74,75,76,77,78,79,80].

According to the literature, palatal dislocations (PDCs) occur significantly more frequently than vestibular dislocations (BDCs). It has been found that eruption disorders of the permanent maxillary canine are closely related to both genetic factors and local conditions. PDCs and BDCs have different aetiologies [81,82,83,84,85,86,87,88].

As for the former (PDC), there are two theories: the guidance theory and the genetic theory. According to the guidance theory, the root of the lateral incisor plays a crucial role in guiding the maxillary canine during its eruption towards the most favorable direction [89,90,91]. If there is excessive space due to a lateral incisor with shape abnormalities or even absent, the canine tends to move palatally [92,93,94,95,96,97].

Genetic theory, on the other hand, suggests that PDCs are a genetically predetermined anomaly with polygenic and multifactorial inheritance [98,99]. The incidence of dental inclusions shows a clear prevalence in females, with an estimated ratio of 2:1 to 3:1 compared to males [100]. This data suggests the existence of a genetic component in the determination of malpositioning, potentially related to sex chromosomes. Conversely, buccal displacement of maxillary canines has been strongly associated with crowding in the arch and therefore a lack of space to accommodate the canine itself [101].

### 1.2. The Importance of CBCT in the Diagnosis of Canine Impaction

The introduction and progressive spread of CBCT in dental imaging has facilitated the study and classification of impacted canines [83,102,103,104,105]. Compared to traditional two-dimensional techniques, CBCT offers clear advantages, allowing three-dimensional reconstruction in all three planes of space [106,107,108,109]. Additional benefits include reduced radiation dose, lower costs and less image distortion compared to other computed tomography methods [110,111,112,113,114,115,116,117].

This technology is used to accurately locate the intraosseous position of the canine and to analyze the main predictive factors for inclusion, such as the inclination relative to the axis of the lateral incisor, the distance from the occlusal plane and the sector to which it belongs. In addition to localisation, CBCT allows the quantity and quality of the alveolar bone to be assessed using the Hounsfield index, calculated on the basis of greyscale values [118,119,120,121,122,123,124]. HUs represent linear transformations of X-ray attenuation coefficients, with conventional reference to water (HU = 0). Although alternative methods have been proposed, such as fractal analysis of bone trabeculae for the study of microstructure, the use of HUs remains the reference method for assessing bone quality in support of impacted teeth [125,126,127,128,129,130,131,132].

Despite the extensive literature addressing the etiological factors associated with maxillary canine impaction—such as genetic predisposition, absence of lateral incisor guidance, arch crowding, and spatial or angular radiographic predictors—the role of the surrounding alveolar bone quality has received comparatively limited attention. In most previous investigations, alveolar bone density has been interpreted primarily as a secondary radiographic finding or as a consequence of failed eruption, rather than as a biological variable that might independently influence the eruptive process.

The advent of cone-beam computed tomography (CBCT) has enabled a more accurate three-dimensional assessment of the periradicular bone environment, allowing quantitative estimation of bone density based on grayscale values commonly expressed as Hounsfield Units (HU). However, the use of HU in CBCT imaging remains a subject of ongoing debate. CBCT-derived voxel values are known to be influenced by device-specific characteristics, acquisition parameters, scatter, beam hardening, and reconstruction algorithms.

In light of this inter-system variability, CBCT-derived HU values should primarily be interpreted as relative measurements, valid within the same scanner and under standardized acquisition conditions. Explicit recognition of this limitation is essential to avoid overinterpretation of absolute values and to ensure methodological transparency. Within this framework, the present study aims to address a gap in the current literature by investigating the association between alveolar bone density and maxillary canine impaction—not to infer causality, but to explore its potential role as an additional biological indicator that may contribute to the understanding of eruption failure and support orthodontic diagnostic decision-making.

However, there is a lack of studies evaluating jawbone quality in included and non-included contralateral canine areas using CBCT [133,134,135,136,137,138].

Approaches to measure bone quality and microstructure, such as trabecular number, fractal dimension analysis, and Hounsfield unit analysis, have been proposed and validated [98,139,140,141,142,143,144,145].

To our knowledge, previous studies have not proposed alveolar process bone density as an aetiological factor in canine impaction. However, understanding the effect of alveolar bone microstructure on the etiology of impacted canines may aid in the diagnosis and treatment of the condition.

It has been observed that bone density can influence orthodontic tooth movement, with an increase in the speed of movement as bone density decreases. This can be demonstrated by the faster tooth movements in children compared to those in adults.

The aim of this study is to investigate a possible association between bone density and canine impaction, using CBCT to measure the Hounsfield index. Such a correlation could prove highly relevant for orthodontists, both during the diagnostic phase and in subsequent treatment planning.

The null hypothesis of the study states that there is no correlation between the bone density of the impacted canine and that of the corresponding, fully erupted contralateral canine, as measured by the Hounsfield index.

## 2. Materials and Methods

This study was carried out at the Dentistry Unit of the University of Bari (Italy) in full accordance with the ethical principles governing clinical research and in compliance with the World Medical Association’s Declaration of Helsinki.

The entire research protocol was reviewed and approved by the Ethics Committee of the University of Bari “Aldo Moro” (Prot. n° 968 dated 1 October 2025).

The clinical data were collected during the period from January 2019 to June 2025, and the study is of a retrospective nature. An initial group of 35 patients was considered for inclusion. To ensure consistency and reliability of the collected data, participants had to meet specific eligibility criteria. The inclusion criteria were:

- Presence of a unilateral impaction of the maxillary canine (experimental group);
- Spontaneous eruption of the contralateral canine (control group);
- Patients still in the growth phase, with all teeth either fully erupted in the arch;
- No history of facial trauma, systemic disease, facial or dental asymmetries, periapical or periradicular lesions, or periodontal/endodontic radiolucencies;
- No vertical or horizontal loss of alveolar bone.

Nine patients who did not meet these criteria were excluded, leaving a final sample of 26 patients—10 males and 16 females—aged between 13 and 19 years. Among the impacted canines analyzed, 12 were palatally positioned and 14 buccally positioned.

**Figure 2:**
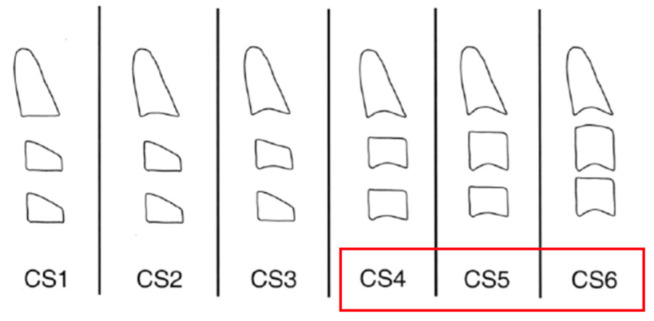
Schematic representation of the stages of maturation of the cervical vertebrae. The red box indicates the stages found in the patients included in the study.

Skeletal maturity was assessed through analysis of cervical vertebral maturation, a widely recognized and commonly used method in orthodontics for estimating a patient’s stage of growth. In the present sample, the stages ranged from C3 to C6, which correspond to a period extending from the onset of the pubertal growth spurt to roughly two years afterward—a particularly important phase for studying facial growth processes and dento-skeletal development (Figure 2).

For each of the 26 patients, CBCT scans taken before any orthodontic treatment were collected and analyzed using a Sirona scanner.

Before image acquisition, patients were seated upright with the head positioned vertically, ensuring that the intersecting reference lines were properly aligned both horizontally and vertically with the center of the region of interest.

All CBCT images analyzed in the present study were acquired using a Sirona GALILEOS scanner (Bensheim, Germany). with standardized acquisition parameters for all patients. Specifically, scans were performed at a tube voltage of 120 kVp and a tube current of 47 mA, with a voxel resolution of 250 μm and a field of view (FOV) of 16 cm. The use of a single imaging device and uniform acquisition parameters limited inter-system variability, allowing bone density values to be interpreted as relative intra-scanner measurements, in accordance with current recommendations regarding the use of CBCT-derived HU values.

**Figure 3:**
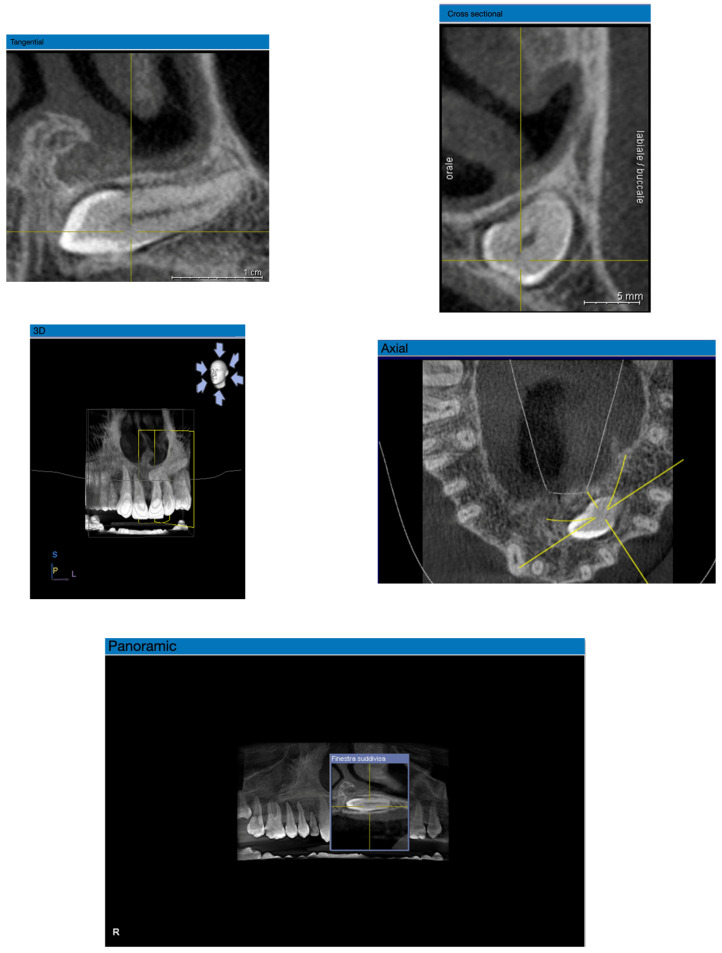
Representation of the five display windows.

To analyze the CBCT images, the Sirona GALILEOS Viewer Software (Version 1.9) medical imaging software was used. This program provides five display windows: panoramic, tangential, 3D, axial, and cross-sectional views (Figure 3).

Canine localization was performed using panoramic and three-dimensional images, while bone density was assessed on the axial sections. In cases where the canines were positioned particularly horizontally within the bone, the cross-sectional view was also employed.

**Figure 4:**
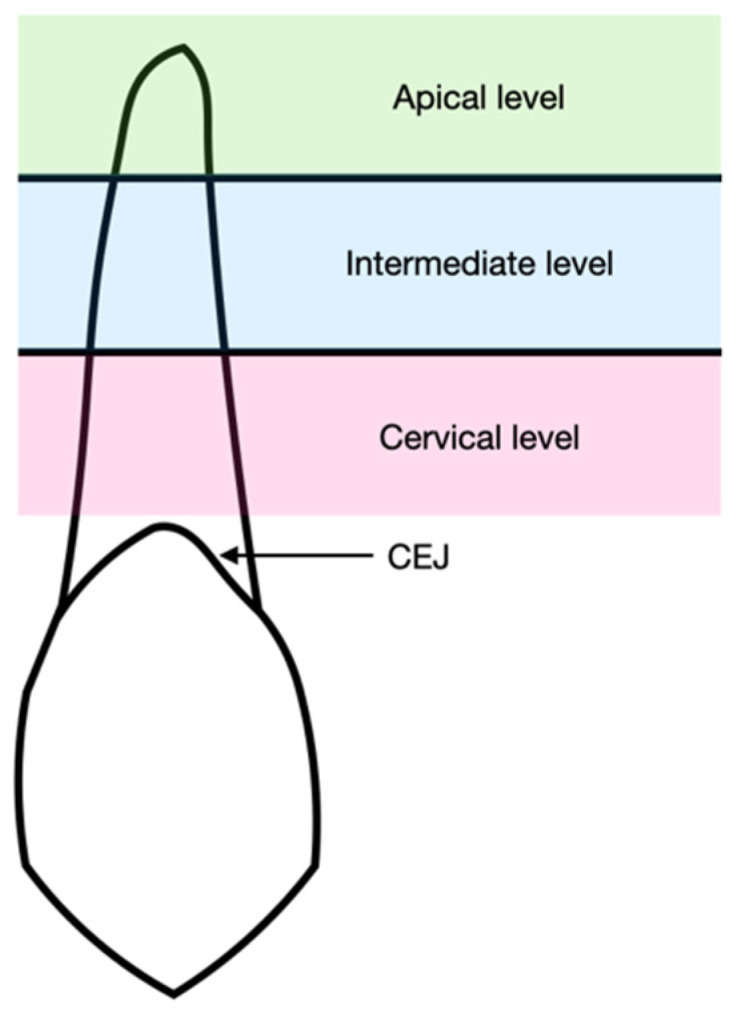
Graphical representation of the three portions of the root on which bone density was assessed.

Bone density was measured using the Hounsfield Unit (HU) index, which directly reflects the tissue attenuation coefficient. For each tooth, bone density was evaluated at three levels (Figure 4):

- Cervical, located 3 mm apical to the cemento-enamel junction (CEJ);
- Intermediate, located 8 mm apical to the CEJ;
- Apical, located 1 mm coronal to the root apex.

Although CBCT provides a three-dimensional volumetric dataset, bone density measurements were performed on standardized two-dimensional axial sections extracted from the reconstructed volume. The region of interest was first expanded by 1 voxel to include the thickness of the periodontal ligament and subsequently by an additional 3 voxels to encompass the surrounding alveolar bone. Tooth and periodontal ligament voxels were excluded from the analysis by subtracting the combined tooth–PDL area from the region of interest, thereby ensuring that only alveolar bone grayscale values were considered.

## 3. Statistic Analysis

Statistical analysis was performed using IBM^®^ SPSS^®^ Statistics software, version 20. Descriptive statistics were calculated for all continuous variables and expressed as means and measures of dispersion. Given the exploratory nature of the study and the paired design, parametric analyses were applied to compare bone density values between impacted canines and their spontaneously erupted contralateral counterparts.

Student’s *t*-test was used to assess differences in mean bone density values across the investigated sites and root levels. Linear regression and correlation analyses were conducted to evaluate the relationship between bone density and patient-related variables, specifically age and sex, with bone density considered as the dependent variable. Correlation coefficients were used to describe the strength and direction of the observed associations.

Statistical significance was set at *p* < 0.05, and all tests were two-tailed. In interpreting the results, statistical significance was considered alongside the magnitude and consistency of the observed differences, in order to support a clinically meaningful interpretation of the findings within the limits of a cross-sectional design.

## 4. Results

Our sample consisted of 26 patients, divided into 16 females and 10 males, aged between 13 and 19 years.

Analysis of the sample’s X-rays showed that the tooth most frequently affected by impaction was 2.3 (Table 1), with a prevalence of 61.5%, presenting a palatal location in 53.8% of cases (Table 2).

The average bone density values were measured at the cervical, mid and apical levels in both the impacted canine and the erupted canine in the arch.

The average bone density in impacted canines decreases from approximately 1190 HU at age 13 to 1603.3 at age 19, while in erupted canines it varies from 1087.3 HU at age 13 to 1270 HU at age 19 (Table 3). More specifically: at 14 years of age, the average values are 1296.7 HU (impacted canine) and 1081.9 HU (erupted canine); at 15 years of age, 1463.3 HU (impacted canine) and 1214.8 HU (erupted canine); at 16 years of age, 1433.8 HU (included canine) and 1140.56 HU (erupted canine); at 17 years of age, 1482.5 HU (included canine) and 1205.5 HU (erupted canine); at 18 years of age, 1573.8 HU (impacted canine) and 1252 HU (erupted canine).

**Figure 5:**
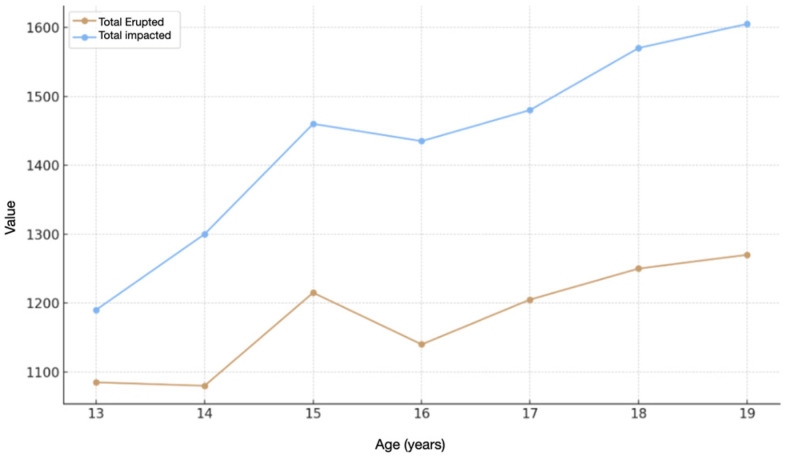
Graphical representation of the relationship between patient age and alveolar bone density in impacted and erupted canines, showing the age-related trend across the analyzed root levels.

There was a statistically significant increase in bone density in impacted canines at all levels analyzed (cervical, intermediate and apical) (Figure 5).

Specifically, the correlation between patient age and bone density showed a statistically significant progressive increase in density in the apical direction in impacted canines. In erupted canines, however, the increase observed did not reach statistically significant values.

To improve the clarity and interpretability of data presentation, mean bone density values observed in impacted canines and contralateral erupted canines were evaluated while also considering their variability. Across all analyzed sites and root levels, the increase in mean bone density values in impacted canines compared with contralateral erupted teeth remained consistent, with data dispersion compatible with the observed statistical significance.

Analysis of the association between age and bone density revealed a positive correlation in impacted canines, indicating a progressive increase in bone density with increasing patient age, whereas this trend did not reach statistical significance in erupted canines. This relationship was particularly evident at the apical level, where bone density showed the greatest increase. The observed correlations support the graphical findings and confirm an age-dependent trend specific to impacted sites.

**Figure 6:**
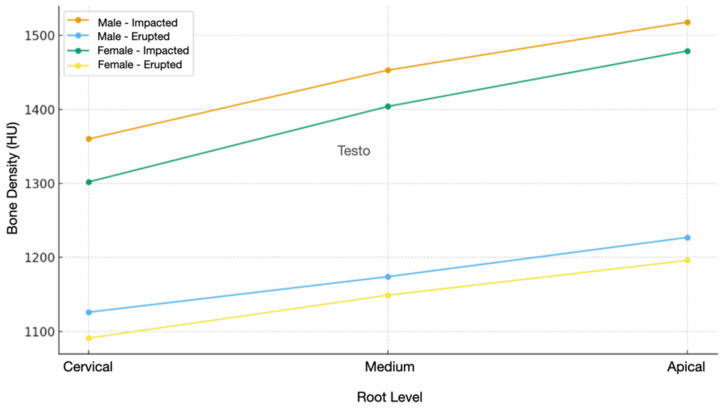
Comparison of mean alveolar bone density values between male and female patients for impacted and erupted canines at different root levels.

In addition, the correlation between bone density and the sex of the patient affected by inclusion was evaluated. Figure 6 shows that in male subjects, the average was 1360 HU at the apical level, 1453 HU at the mid level, and 1518 at the apical level in the impacted tooth; in the erupted tooth, the average was 1126 HU at the apical level, 1174 HU at the mid level, and 1227 at the apical level. In female subjects, there is an average of 1302 HU at the apical level, 1404 HU at the mid level, and 1479 at the apical level in the impacted tooth; in the erupted tooth, there is an average of 1091 HU at the apical level, 1149 HU at the mid level, and 1196 at the apical level.

## 5. Discussion

Impaction of the maxillary canine is among the most frequent and challenging problems in orthodontics, with an estimated prevalence of about 1–3% [27,143,144,145,146]. Early diagnosis and careful treatment planning are crucial—not only to preserve esthetics and occlusal function, but also to prevent complications such as root resorption or loss of periodontal support [147,148,149,150,151,152].

Three-dimensional imaging, particularly cone-beam computed tomography (CBCT), has become an essential diagnostic tool, as it allows accurate localization of the impacted tooth and provides detailed information about the surrounding bone. One of the most valuable parameters is bone density expressed in Hounsfield Units (HU), which helps clarify the biological mechanisms behind impaction and guides the design of a more predictable orthodontic–surgical plan [94,149].

The results of this investigation contribute to a growing interest in understanding how the quality of the surrounding alveolar bone may influence, or be influenced by, the impaction process. Although several radiographic indicators—such as angle α, distance d, and the sector classification—have long been used to estimate the complexity of canine traction, the potential diagnostic value of CBCT-derived bone density has received far less attention [89].

In the present analysis, the bone surrounding impacted canines displayed consistently higher density values than that around the contralateral erupted teeth, regardless of the level or surface considered. This uniform trend suggests that the bone environment at impaction sites may undergo reduced turnover and progressive mineralization, likely due to the absence of eruptive forces. Such a pattern could act not only as a by-product of prolonged impaction but also as a barrier that further limits the tooth’s ability to migrate toward the arch.

The results of the present study should be interpreted within the context of the still limited number of investigations that have employed CBCT to assess alveolar bone density in orthodontics. Previous studies have shown that bone density values may vary in relation to anatomical, functional, and developmental factors, thereby influencing the biological response to tooth movement. In line with these observations, our findings demonstrate a consistent increase in bone density in impacted areas compared with contralateral erupted sites; however, the cross-sectional nature of the study design does not allow determination of whether this increase represents a predisposing factor for impaction or rather a consequence of prolonged absence of eruptive stimuli. It is plausible that both conditions coexist within a bidirectional mechanism, whereby increased bone density may initially hinder eruption and, at the same time, progressively increase as a result of tooth impaction. This integrated interpretation allows the findings of the present study to be positioned coherently within the existing literature, avoiding excessive causal inference and reinforcing the biological and clinical relevance of the observed association.

Comparison within the same patient reinforces the idea that this increase in density is a localized phenomenon rather than a general consequence of age-related skeletal changes. Moreover, although canine impaction is more frequent in females, sex-related variations in bone density were minimal, indicating that sex does not substantially affect the bone microenvironment once impaction has occurred.

Denser bone—compatible with D1–D2 quality—may hypothetically hinder orthodontic biomechanics by reducing the rate and predictability of tooth movement; however, this interpretation should be regarded as speculative, as no direct treatment outcome data were available in the present study. These findings highlight the need for individualized treatment planning, which in some cases may require adjunctive procedures aimed at enhancing bone remodeling [119].

From a clinical standpoint, early detection of unusually dense alveolar bone through CBCT may hypothetically help clinicians anticipate potentially longer or more complex treatment courses; however, this consideration is based on biological plausibility rather than on direct clinical outcome evidence from the present sample.

CBCT thus offers not only superior 3D localization of the impacted tooth but also a more comprehensive assessment of the biological environment influencing treatment success.

Overall, the results point to alveolar bone density as a parameter worthy of integration into the diagnostic workflow for impacted canines. While causal relationships cannot be determined from cross-sectional data, the consistent association observed warrants further longitudinal research to clarify how bone density evolves over time and how it may change following orthodontic–surgical intervention.

## 6. Limitations

Despite its clinical relevance, this study has some limitations. The relatively small sample size reduces the statistical power and makes it more difficult to generalize the findings.

An additional limitation of the present study is the absence of a stratified analysis of bone density according to the direction of maxillary canine impaction (palatal vs. buccal). Although the overall sample included both types of impaction, subdividing the cohort into smaller subgroups would have resulted in a substantial reduction in sample size for each group, with an increased risk of statistically unstable and difficult-to-interpret results. Given the exploratory nature of the study and its primary objective of assessing the association between alveolar bone density and maxillary canine impaction through an intra-subject comparison with the contralateral erupted canine, it was considered methodologically more appropriate to focus the analysis on the overall effect of impaction, regardless of its direction. Stratified analyses according to impaction direction would require larger samples and specifically designed studies to ensure adequate statistical power and reliable interpretation of the results and therefore represent an important avenue for future research.

An additional limitation of the present study is that intra- and inter-observer reliability of bone density measurements was not formally assessed. Although all measurements were performed using a standardized protocol and clearly defined anatomical reference points, the lack of reproducibility analysis may have introduced an unquantified degree of measurement variability. Future studies should include specific reliability assessments to further strengthen the methodological robustness of CBCT-based bone density evaluations.

An additional limitation of the present study concerns the intrinsic characteristics of CBCT-derived bone density measurements. As discussed in previous sections, HU values obtained from CBCT should be interpreted as relative measurements, as they are influenced by the imaging device, acquisition parameters, and reconstruction algorithms, and are not directly comparable with Hounsfield Units derived from multidetector computed tomography. Furthermore, the cross-sectional design of the study and the lack of longitudinal follow-up preclude causal inference and do not allow determination of whether the increased bone density observed represents a predisposing factor for tooth impaction or rather a consequence of prolonged eruption failure. Future longitudinal studies, conducted on larger samples and using standardized protocols, will be necessary to further clarify these aspects.

Future research with larger cohorts and preferably prospective follow-up after orthodontic–surgical treatment will be needed to confirm these results and to track how bone density changes over time and how it relates to the timing and success of canine recovery.

## 7. Conclusions

The results of the present study demonstrate an association between increased alveolar bone density and maxillary canine impaction, with higher bone density values observed in impacted areas compared with contralateral spontaneously erupted sites. This association appears to be more evident in relation to patient age and at more apical root levels.

Given the cross-sectional nature of the study design and the intrinsic limitations of CBCT-derived bone density measurements, these findings do not allow causal inferences to be drawn. Accordingly, alveolar bone density should not be interpreted as a definitive etiological determinant of tooth impaction, but rather as a complementary diagnostic parameter that may contribute to a more comprehensive assessment of the local biological environment.

In this context, integrating bone density information with other clinical and radiographic indicators may support orthodontic diagnostic evaluation and treatment planning. Future studies, preferably longitudinal and conducted on larger patient cohorts, are warranted to further clarify the role of alveolar bone density in the dental eruption process.

## Figures and Tables

**Table 1:** Frequency of impacted canines per side (right/left).

	Frequency	Percentage %
**Upper right canine impacted (1.3)**	10	38.5%
**Lower left canine impacted (2.3)**	16	61.5%
**Total**	26	100%

**Table 2:** Distribution of impacted canines based on position (vestibular/palatal).

	Frequency	Percentage %
**Vestibular Inclusion**	12	46.2%
**Palatal Inclusion**	14	53.8%
**Totale**	26	100%

**Table 3:** Correlation between patient age and alveolar bone density values in impacted and contralateral erupted canines, expressed as mean values at different root levels.

Age	Apical Erupted	Medium Erupted	Cervical Erupted	Apical Impacted	Medium Impacted	Cervical Impacted	Total Impacted	Total Erupted
Mean (13 Years)	1161.3	1081.4	1019.2	1409	1333.1	1180	1190.05	1087.3
Patients (13 Years)	4	4	4	4	4	4	4	4
SD (13 Years)	103.2	106.7	105.2	117.7	134.6	155	133.5	105.5
Mean (14 Years)	1119.3	1089.1	1037.4	1396.6	1294.4	1199.6	1296.7	1081.9
Patients (14 Years)	4	4	4	4	4	4	4	4
SD (14 Years)	97.2	88.3	64.7	93.9	63.7	87.5	67.1	81.15
Mean (15 Years)	1259.5	1221.4	1163.5	1541.5	1481.4	1368.3	1463.3	1214.8
Patients (15 Years)	6	6	6	6	6	6	6	6
SD (15 Years)	41.2	65.4	69.3	99.8	98.3	103.1	94.28	53.96
Mean (16 Years)	1193.1	1131.3	1097.3	1510.4	1443.5	1347.6	1433.8	1140.56
Patients (16 Years)	4	4	4	4	4	4	4	4
SD (16 Years)	113.3	105.2	120.3	174.5	196.4	184.3	182.21	108.03
Mean (17 Years)	1261	1210.3	1145.4	1547.3	1499.2	1401.2	1482.5	1205.5
Patients (17 Years)	3	3	3	3	3	3	3	3
SD (17 Years)	76.5	57.1	52.3	83.4	90.1	105.5	90.49	60.3
Mean (18 Years)	1301.5	1251.3	1203.2	1633.4	1567	1521	1573.8	1252
Patients (18 Years)	3	3	3	3	3	3	3	3
SD (18 Years)	74.7	97.3	107.4	43.6	42.3	63.4	46.95	89.8
Mean (19 Years)	1320	1270	1220	1660	1600	1550	1603.3	1270
Patients (19 Years)	2	2	2	2	2	2	2	2
SD (19 Years)	65	70	80	50	45	60	55	75

## Data Availability

The data presented in this study are not publicly available due to privacy and ethical restrictions, as they consist of retrospective clinical radiographs and patient records. Anonymized data may be made available from the corresponding author upon reasonable request and pending approval by the institutional ethics committee.
